# Maternal UHRF1 Is Essential for Transcription Landscapes and Repression of Repetitive Elements During the Maternal-to-Zygotic Transition

**DOI:** 10.3389/fcell.2020.610773

**Published:** 2021-02-09

**Authors:** Yanqing Wu, Juan Dong, Shenglei Feng, Qiang Zhao, Peng Duan, Mengneng Xiong, Yujiao Wen, Chunyu Lv, Xiaoli Wang, Shuiqiao Yuan

**Affiliations:** ^1^Tongji Medical College, Institute Reproductive Health, Huazhong University of Science and Technology, Wuhan, China; ^2^Department of Obstetrics and Gynecology, Union Hospital, Tongji Medical College, Huazhong University of Science and Technology, Wuhan, China; ^3^Central Laboratory, Xiangyang No.1 People's Hospital, Hubei University of Medicine, Xiangyang, China; ^4^Laboratory of Gynecological Oncology and Reproductive Health, Department of Obstetrics and Gynaecology, Xiangyang No.1 People's Hospital, Hubei University of Medicine, Xiangyang, China; ^5^Shenzhen Huazhong University of Science and Technology Research Institute, Shenzhen, China

**Keywords:** UHRF1, maternal-to-zygotic transition, H3K4Me3, 5mC, retrotransposon

## Abstract

Maternal factors that modulate maternal-to-zygotic transition (MZT) are essential for the growth from specialized oocytes to totipotent embryos. Despite several studies, the mechanisms regulating epigenetic reprogramming during MZT remain largely elusive. UHRF1 plays a role in maintaining GC methylation in oocytes and early embryos. However, little is known about its role in mouse MZT. Here, we explored the function of maternal UHRF1 in zygotic genome regulation during early embryonic development in mice. We showed that the conditional knockout (cKO) of UHRF1 in either primordial or growing oocytes causes infertility but differentially affects early embryonic development. UHRF1 deficiency in primordial oocytes led to early embryonic developmental arrest at the two-cell stage, accompanied by significant alterations in global DNA and H3K4me3 methylation patterns. In comparison, UHRF1 ablation in growing oocytes significantly reduced developmental competence from two-cell embryos to blastocysts. At the transcriptional level, the absence of maternal UHRF1 led to aberrant transcriptional regulation of the zygotic genome during MZT at the two-cell stage. Furthermore, we observed that retrotransposable elements in UHRF1-deficient oocytes and embryos were not silenced properly; in particular, the LINE-1 and long terminal repeat (LTR) subfamily were activated abnormally. Collectively, the findings of our study reveal that maternal UHRF1 plays a critical role in establishing the correct epigenetic chromatin reprogramming of early embryos, regulating essential genes during MZT, and preserving genome integrity that drives early embryonic development in mice.

## Introduction

Fully grown mammalian oocytes are highly differentiated and transcriptionally quiescent. Upon fertilization, early embryonic development involves the “maternal-to-zygotic transition (MZT),” in which the majority of the maternal RNAs and proteins are degraded, the zygotic genome becomes transcriptionally active, and embryos are totipotently established (Piko and Clegg, 1982; Hamatani et al., 2004; Tadros and Lipshitz, 2009; Walser and Lipshitz, 2011). In mice, zygotic genome activation (ZGA) is a key event of in MZT (Newport and Kirschner, 1982; Tadros and Lipshitz, 2009; Lee et al., 2014) and occurs in two phases: minor ZGA initiates during the late zygotic stage and major ZGA follows at the two-cell embryo stage (Kanka, 2003; Artus and Cohen-Tannoudji, 2008; Xue et al., 2013). In addition, ZGA is characterized by efficient TATA-less promoters use (Davis and Schultz, 2000), activation of retrotransposons (Peaston et al., 2004), for example, murine endogenous retrovirus with a leucine tRNA primer-binding site (MERVL) at the two-cell stage as a marker for totipotent cells (Macfarlan et al., 2012), transcription and translation uncoupling in zygotes (Nothias et al., 1996), and transcription activation in two-cell embryos (Wiekowski et al., 1991). Transcription from the maternal pool is critical for ZGA and embryonic development. Defects in ZGA initiation always lead to embryonic development failure, in which embryos are mainly arrested at the two-cell stage and cannot develop into totipotent blastocysts (Qiu et al., 2003; Chu et al., 2013; Zhang et al., 2015). Thus, illustrating the key maternal ZGA regulators will facilitate the understanding of early embryogenesis.

DNA methylation and histone modifications are essential for ZGA (Guo et al., 2014). For example, 5-methylcytosine (5-mC) and 5-hydroxymethylcytosine (5-hmC), as a stable epigenetic modification, play important roles during early embryonic development (Stroud et al., 2011; Bachman et al., 2014). In addition, histone modification, including lysine acetylation and lysine (tri-) methylation, is required for mouse embryogenesis during ZGA. Loss of the maternal BRG1, a component of ATP-dependent chromatin remodeling by acetylation, results in reduced expression levels of zygotic genes and early embryo arrest at the two-cell stage (Bultman et al., 2006). The opposing marks histone H3 trimethylated at lysine 4 (H3K4me3) and histone H3 lysine 27 trimethylation (H3K27me3) are associated with gene activation and repression, respectively. Upon fertilization, H3K4me3 and H4 acetylation are re-established on promoter regions during the major ZGA at the late two-cell stage (Liu et al., 2016; Zhang et al., 2016). Endogenous retroviruses (ERVs), which were previously considered to be “junk DNA,” are broadly transcribed into tissue-specific genes or ERV-derived sequences in early embryos (Kigami et al., 2003; Peaston et al., 2004; Bui et al., 2009). ERVs expression contributes to embryonic genome activation and totipotency and pluripotency establishment (Lu et al., 2014). UHRF1 (ubiquitin-like, containing PHD and RING finger domains1), an epigenetic factor and ubiquitin ligase, is a key epigenetic regulator between DNA methylation maintenance and histone modifications in both somatic and male germ cells (Bostick et al., 2007; Xie et al., 2012; Liu et al., 2013; Dong et al., 2019). Two recent studies have revealed that maternal UHRF1 is essential for oocyte and preimplantation embryo development by regulating several epigenetic pathways, and maternal UHRF1 deficiency in growing oocytes induces early embryonic developmental defects (Maenohara et al., 2017; Cao et al., 2019). However, the role of maternal UHRF1 in the epigenetic regulation of the MZT process has not yet been explored.

In this study, we deleted UHRF1 in oocytes at two different developmental stages: primordial and growing oocytes. Both conditional knockouts (cKOs) caused infertility, but displayed different early embryonic development defects, demonstrating the crucial role of maternal UHRF1 in reproduction. Our study, for the first time, showed that maternal UHRF1 depletion in primordial oocytes results in more severe embryo arrest phenotype than that in growing oocytes. The absence of UHRF1 protein in primordial oocytes led to early embryonic developmental arrest at the two-cell stage, with a severe and stepwise decrease in H3K4me3 levels at zygotes and two-cell stage embryos. Furthermore, the absence of UHRF1 in primordial oocytes abrogated the normal changes in transcriptome by the two-cell stage, resulting in the deficient suppression of retrotransposon expression and increased genome damage, possibly due to increased LINE-1 activity. Altogether, our study demonstrated the essential role of maternal UHRF1 in early embryonic development during MZT and provided new insight into the maintenance of appropriate temporal and spatial patterns of histone methylation and preserving genome expression and integrity to ensure MZT and developmentally competent in preimplantation embryos.

## Materials and Methods

### Mice

Floxed *Uhrf1* mice (*Uhrf1*^*flox*/*flox*^) were obtained from Shanghai Research Center for Model Organisms, and the details were referred to in the previous article (Dong et al., 2019). *Zp3-Cre* and *Gdf9-Cre* in the C57BL/6J background was purchased from the Jackson Laboratory. *Zp3-Cre* or *Gdf9-Cre* males were first crossed with *Uhrf1*^*flox*/*flox*^ females to generate the *Zp3-Cre*; *Uhrf1*^+/*flox*^ or *Gdf9-Cre*; *Uhrf1*^+/*flox*^ females, and then the *Zp3-Cre*; *Uhrf1*^+/*flox*^ or *Gdf9-Cre*; *Uhrf1*^+/*flox*^ female mice were bred with *Uhrf1*^*flox*/*flox*^ male mice to obtain the *Zp3-Cre*; *Uhrf1*^*flox*/*lox*^ or *Gdf9-Cre*; *Uhrf1*^*flox*/*lox*^ females (designated as *Zp3-*cKO or *Gdf9*-cKO). For the genotype, the primers of PCR are listed in Supplementary Table 1.

### Antibodies

All the commercial antibodies in the study are listed in Supplementary Table 2. The antibody of LINE1 ORF1 antisera generation was homemade, which was the same with the one used in our previous study.

### Oocytes and Early Embryos Collection and Culture

For the superovulation, each 8- to 10-week-old female mouse was injected with 10 IU PMSG, followed by 10 IU hCG after 48 h. The cumulus–oocyte complexes were collected from the oviducts at 16 h of hCG injection. After the 0.5 mg/ml hyaluronidase (Sigma, St. Louis, MO, USA) treatment, MII oocytes without cumulus cells were collected. To obtain the zygotes, female mice were mated with 8- to 10-week-old WT male mice. After the presence of vaginal plugs being found, embryos were harvested from oviducts at 16 h after hCG injection. To obtain the developmental embryos, zygotes were cultured in KSOM medium for differenced time. After the culture, zygotes, two-cell embryos, four-cell embryos, eight-cell embryos, morula, and blastocysts were collected for additional experiments.

### Hematoxylin and Eosin (HE) Staining and Immunohistochemistry (IHC)

Mouse ovaries were collected and fixed in Bouin's solution (SIGMA, HT10132) at 4°C overnight and then washed five times with 75% alcohol, 30 min per wash. After washing with 100% alcohol and xylene penetration for 2 h, ovaries were embedded in paraffin, and 8-μm-thick sections were serially cut and stained with HE after being dewaxed and rehydrated. To determine the number of different types of follicles per ovary, every fifth section was counted throughout the whole ovary from the first section to the final count and the total number was multiplied by five as a correction factor, which is based on the well-accepted standards established by Pedersen and Peters (1968). Briefly, after the slices were sectioned, the number of follicles was counted by the well-accepted criteria established by Pederson and Peters. The primordial follicles (types 1 and 2), primary follicles (type 3), early secondary and late secondary follicles (types 4 and 5), and antral follicles (types 6 and 7) were counted in collected sections of an ovary. In each section, only those follicles that contain the nucleus of the oocyte were scored. The number of the follicle counts was multiplied by a correction factor of 5 to represent the estimated number of total follicles in an ovary. After being dewaxed and rehydrated, the sections for IHC were microwaved with 0.01% citrate (pH 6.0) and cooled at room temperature (RT). After washing three times with PBS, the sections were treated with 3% H_2_O_2_ at RT for 15 min. After blocking with 5% BSA for 1 h, the sections were incubated with primary antibody at a 4°C wet box overnight. Then, the sections were washed with PBS and incubated with a secondary antibody for 1 h at RT. After washing with PBS and coloring with DAB, the sections were stained with hematoxylin and washed with ddH_2_O. After hydrating, the sections were mounted with neutral resin and then photographed.

### Immunofluorescence Staining

For oocyte and embryo immunofluorescence staining, oocytes, and embryos were fixed in 4% paraformaldehyde (PFA) for 30 min at RT and then permeabilized for 20 min in 0.1% Triton X-100 in PBS at RT. After blocking in 1% BSA in PBS at RT for 1 h, the oocytes and embryos were incubated with primary antibody at 4°C overnight. The oocytes and embryos were washed three times in 1 mg/ml polyvinyl pyrrolidone (PVP; Sigma) in PBS. After incubation with secondary antibody for 1 h at RT, the oocytes and embryos were stained with DAPI in the dish and examined by FluoView 400 microscope (Zeiss, Germany). The fluorescence intensity measurement on immunofluorescence Z stacks was performed by Image J software. The process is as follows: the nuclear area of the stack image was selected, and then the integrated Intensity (intensity divided by the number of pixels represented within the nuclear area) was obtained. Distribution of fluorescence intensities (background color removed) was compared using *t* test after all data had been tested as belonging to normally distributed populations (SPSS19.0 software).

### Western Blotting

Oocytes were collected from WT and mutant mice, and the proteins were extracted using RIPA buffer (CWBIO, Cat# 01408). The protein lysates of 100 oocytes were separated on a 10% SDS-PAGE gel and transferred to PVDF membranes (Bio-Rad). After 5% non-fat milk blocking for 1 h, the membranes were incubated with primary antibodies overnight at 4°C. After washing three times with TBST, the membranes were then incubated with the secondary antibodies for 1 h at RT. After washing, the membranes were chemiluminescence detected and photographed by ChemiDoc XRS+ system (BIO-RAD).

### RNA Isolation and RT-qPCR

Total RNAs were extracted from oocytes or embryos using TRIzol reagent (Life Technologies) according to the manufacturer's protocols. The purity and concentration of RNA samples were determined by the Nanodrop ND-2000 spectrophotometer (Thermo Scientific). A total of 500 ng of RNA was reverse transcribed according to the High Capacity cDNA Reverse Transcription Kit (Thermo Scientific) to obtain cDNAs. RT-qPCR was performed with SYBR green master mix (Vazyme) on the StepOne Real-Time PCR system according to manufacturer's instructions. Each experiment was performed in triplicate using the comparative cycle threshold method, with the *Gapdh* expression used as normalization. The specific primers are listed in Supplementary Table 1.

### RNA-Seq Analysis

RNAs (300 ng) from MII oocytes and two-cell embryos were used for stranded RNA sequencing library preparation. After the mRNA was enriched with Oligo(dT) magnetic beads, the fragmentation buffer was added to break the mRNA into short fragments, and the mRNA was used as a template to synthesize one-stranded cDNA using random hexamers. The purified double-stranded cDNA was subjected to plus A-tail and ligated with sequencing joints before being fragmented using AMPure XP beads size selection. The second strand of the U-containing cDNA is degraded by USER enzyme, and then the final sequencing information is derived from the first strand of cDNA, thus preserving the strand orientation of the mRNA. Then, the PCR amplification was performed, and the PCR product was purified with AMPure XP beads to obtain a strand-specific cDNA library. The EDGER package (version 3.12.1) was used to annotate and identify differentially expressed genes (DEGs) among different groups based on the UCSCMM10 mouse genome with the criteria of the FDR corrected *p* value to 0.05 and fold-change cutoff of 2.0. The Gene Ontology (GO) analysis for DEGs was performed by KOBAS software (version: 2.1.1) with a corrected *p* value cutoff of 0.05 to judge statistically significant enrichment.

### RNA-seq Analysis of Repetitive Elements

All the raw sequences of RNA-seq were annotated based on Repbase (http://www.girinst.org/repbase/) with HiSAT2 software. The differentially expressed repetitive elements between control and mutant oocytes and two-cell embryos were collected from the sorted bam file with the Samtools function “idxstats” with the criteria of more than twofold change and Wald test (FDR < 0.05).

### Data Processing and Analysis of ChIP-seq

ChIP-seq reads were downloaded from (GSE113915) datasets. After the read count was normalized, the read count was mapped to the Mouse Genome Overview GRCm38 assembly (USCS mm10) (http://ftp.ensembl.org/pub/release87/fasta/mus_musculus/dna/) using STAR (https://github.com/alexdobin/STAR/ releases). For UHRF1, H3K9me3, and H3K4me3 occupancy in *Cdk1, Cdk2, Dppa2, Dppa3*, and *Dppa4* genes, peak callings were performed by MACS2 (https://github.com/taoliu/MACS), and the default parameters with broad peak option and a broad cutoff of 0.05 (*p* value) were used.

### Statistical Analysis

All data are presented as mean ± SEM. Statistical differences between datasets were assessed by one-way ANOVA or Student's *t* test using the SPSS19.0 software or GraphPad Prism 8.0. *p* values are denoted in figures by ^*^p < 0.05.

## Results

### UHRF1 Deficiency in Primordial or Growing Oocytes Results in a Distinct Early Embryonic Development Arrest

**Figure 1:**
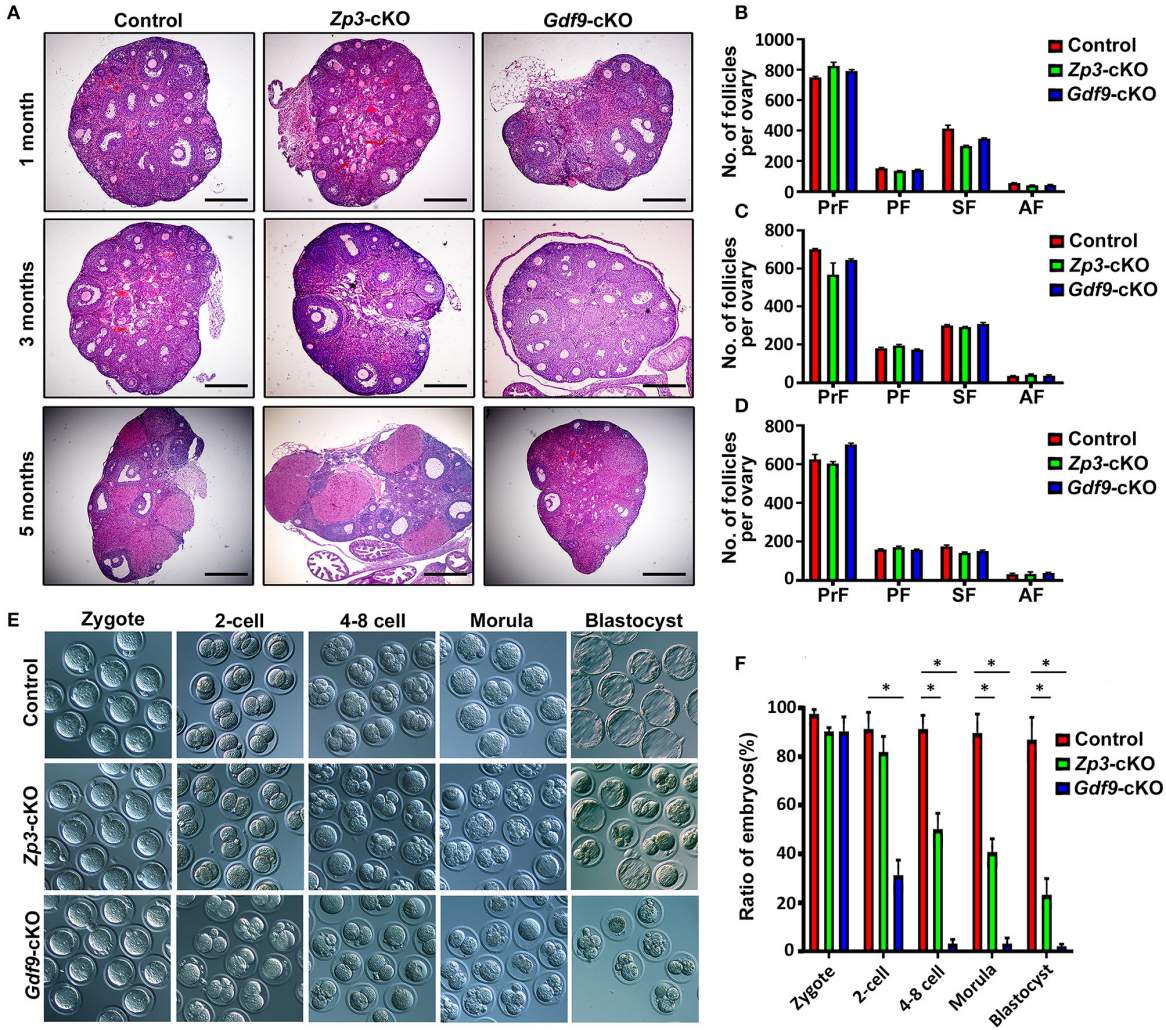
Conditional inactivation of *Uhrf1* in either primordial or growing oocytes results in preimplantation embryo developmental arrest and female sterility in mice. **(A)** Histology of ovarian sections from control, *Zp3*-cKO, and *Gdf9*-cKO mice at 1, 3, and 5 months of age, respectively, stained with Hematoxylin and Eosin. Scale bars = 500 μm. **(B–D)** Quantitative analyses show the number of primordial follicles (PrF), primary follicles (PF), secondary follicles (SF), and antral follicles (AF) per ovary at the age of 1, 3, and 5 months, respectively. *n* = 3 for each genotype; data are presented as mean ± SEM. **(E)** Representative bright-field images show the morphology of the zygotes, two-cell embryos, four- to eight-cell embryos, morula, and blastocysts from control, *Zp3*-cKO, and *Gdf9*-cKO mice. **(F)** Statistical analysis of early embryos before implantation at each developmental stage. Data are presented as means ± SEM from three independent experiments. *p < 0.05.

Since previous reports have shown that UHRF1 in growing oocytes plays a crucial role in the global epigenetic reprogramming of oocytes and preimplantation embryo development (Maenohara et al., 2017; Cao et al., 2019), we explored the function of UHRF1 in primordial oocytes on oocyte and early embryo development. We thus generated a conditionally knockout *Uhrf1* mouse model in primordial oocytes by crossing *Uhrf1*^*flox*/*flox*^ mice with *Gdf9-Cre* mice (Lan et al., 2004) (Supplementary Figures 1A,B). Molecular analyses showed that UHRF1 mRNA and protein were undetectable in *Uhrf1*^*flox*/*flox*^: *Gdf9-cre* mouse oocytes (hereafter referred to as *Gdf9-*cKO) (Supplementary Figures 1C–F), indicating that *Gdf9*-Cre mediated *Uhrf1* knockout mice were successfully created. To confirm and compare the effects of UHRF1 in growing oocytes on female fertility, we also successfully generated *Zp3*-Cre mediated *Uhrf1* knockout mouse models (hereafter referred to as *Zp3*-cKO) as previously reported (Maenohara et al., 2017; Cao et al., 2019) (Supplementary Figures 1A,B,G,H). As expected, no pups were produced in either *Zp3*-cKO or *Gdf9*-cKO females after mating with fertility-proven males for at least 6 months (data not shown), indicating that *Zp3*-cKO and *Gdf9*-cKO females were sterile. To characterize the phenotype of *Gdf9*-cKO and *Zp3*-cKO females, we first examined the histomorphology of their ovaries. The results revealed that both *Zp3*-cKO and *Gdf9*-cKO ovaries at 1, 3, and 5 months were morphologically and histologically indistinguishable from control ovaries, with the presence of follicles at various stages (Figures 1A–D). The observations indicated that specifically deleting the UHRF1 in either primordial or growing oocytes does not affect follicle development and oogenesis. Next, we investigated whether the fertilization ability of oocytes was affected upon UHRF1 deletion in primordial or growing follicles. To this end, *Zp3*-cKO and *Gdf9*-cKO females were superovulated and mated them with WT males. Examination of the oocytes or embryos obtained at 0.5 dpc oviducts showed that *Zp3*-cKO and *Gdf9*-cKO MII oocytes were fertilizable, and all embryos successfully develop into zygotes (Figure 1E), suggesting that the *Zp3*-cKO and *Gdf9*-cKO oocytes had normal fertilization ability. However, we observed that the embryonic development potency of *Zp3*-cKO and *Gdf9*-cKO zygotes was significantly compromised compared to that of WT controls (Figures 1E,F). In *Zp3*-cKO mice, around half of the embryos were blocked at the two- to four-cell stage, and ~20% embryos developed into blastocysts (Figure 1F), which was consistent with the results of a previous study (Cao et al., 2019). Interestingly, in *Gdf9*-cKO mice, we found that most embryos were arrested at the zygote stage; only ~40% embryos developed into two-cell stages, and almost no embryos developed into the blastocysts (Figure 1F). These results indicate that UHRF1 depletion in primordial oocytes results in more severe early embryonic development defects than that due to UHRF1 deficiency in growing oocytes, which further suggests that UHRF1 may have a distinct function in developing primordial oocytes into growing oocytes.

### Maternal UHRF1 Deletion Causes DNA Hypomethylation and Abnormal H3K4 Methylation Pattern in Oocytes and/or Early Embryos

**Figure 2:**
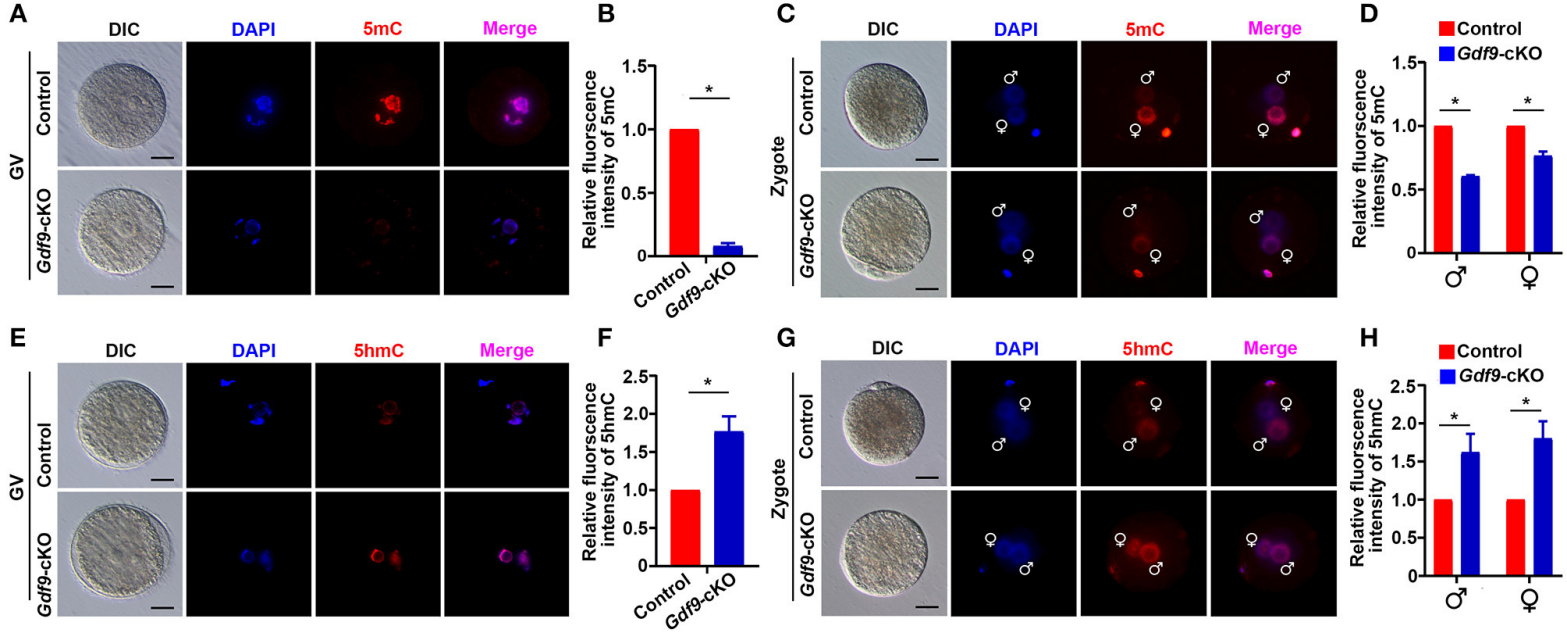
Maternal UHRF1 depletion causes DNA hypomethylation at oocytes and zygotes. **(A)** The representative immunofluorescent-staining images for anti-5-methylcytosine (anti-5-mC) of control and *Gdf9*-cKO GV oocytes are shown. Nuclei were stained with DAPI. Scale bar = 50 μm. **(B)** Histogram shows a semi-quantitative analysis regarding changes of 5-mC distribution of fluorescence intensity at GV oocytes of **(A)**. Quantification of the level of 5-mC in control and *Gdf9*-cKO oocytes and data are presented as means ± SEM from three independent experiments (total number of GV oocytes analyzed: *n* = 92 for control, *n* = 81 for *Gdf9*-cKO). *p < 0.05. **(C)** Representative immunofluorescent-staining images for 5-mC of control and *Gdf9*-cKO zygotes are shown. Nuclei were stained with DAPI. Scale bar = 10 μm. **(D)** Histogram shows a semi-quantitative analysis of changes in the fluorescence intensity, indicating the 5-mC distribution in zygotes of **(C)**. Data are presented as mean ± SEM. *n* = 3 independent experiments (total number of zygotes analyzed: *n* = 131 for control, *n* = 73 for *Gdf9*-cKO). *p < 0.05. **(E,F)** Representative immunofluorescent staining images **(E)** and the quantification of fluorescence intensity **(F)** for 5-hydroxymethylcytosine (5-hmC) of control and *Gdf9*-cKO GV oocytes are shown. Data are presented as mean ± SEM. *n* = 3 independent experiments (total number of GV oocytes analyzed: *n* = 121 for control, *n* = 87 for *Gdf9*-cKO). **(G,H)** Representative immunofluorescent-staining images **(G)** and fluorescence intensity quantification **(H)** for 5-hmC of control and *Gdf9*-cKO zygotes are shown. Data are presented as mean ± SEM. *n* = 3 independent experiments (total number of zygotes analyzed: *n* = 95 for control, *n* = 78 for *Gdf9*-cKO). Scale bar = 50 μm. *p < 0.05.

Next, we explored the molecular reason for embryo developmental arrest upon maternal UHRF1 depletion. Since UHRF1 serves as a master epigenetic regulator between DNA methylation and histone modification in male germ cells (Dong et al., 2019), we examined whether DNA methylation and histone modification were affected in UHRF1-deficient oocytes and preimplantation embryos. We mainly focused on the GV oocytes and early embryos derived from *Gdf9*-cKO mice in this study because the phenotype of *Gdf9-*cKO mice was more severe than that of *Zp3-*cKO mice. Thus, we first investigated the effect of maternal UHRF1 deficiency on the establishment of DNA methylation in GV oocytes and zygotes from control and *Gdf9*-cKO mice. Immunofluorescence analysis revealed that the overall 5-mC expression levels were significantly decreased in GV oocytes of *Gdf9-*cKO mice, where ~80% reductions were seen in mutants compared to controls (Figures 2A,B). Additionally, the overall 5-mC expression levels of the paternal and maternal pronuclei in the zygotes of *Gdf9*-cKO mice were decreased with a one- to two-fold change compared with those in the control (Figures 2C,D). As maternal TET3 catalyzes the conversion of 5-mC to 5-hmC for DNA demethylation during oogenesis (Gu et al., 2011), we examined the expression levels of 5-hmC in GV oocytes and zygotes from control and *Gdf9*-cKO mice. We observed that the expression levels of 5-hmC significantly increased in both GV oocyte and zygote of *Gdf9-*cKO mice (Figures 2E–H). These data indicate that maternal UHRF1 is required for the maintenance of global DNA methylation in oocytes and zygotes.

**Figure 3:**
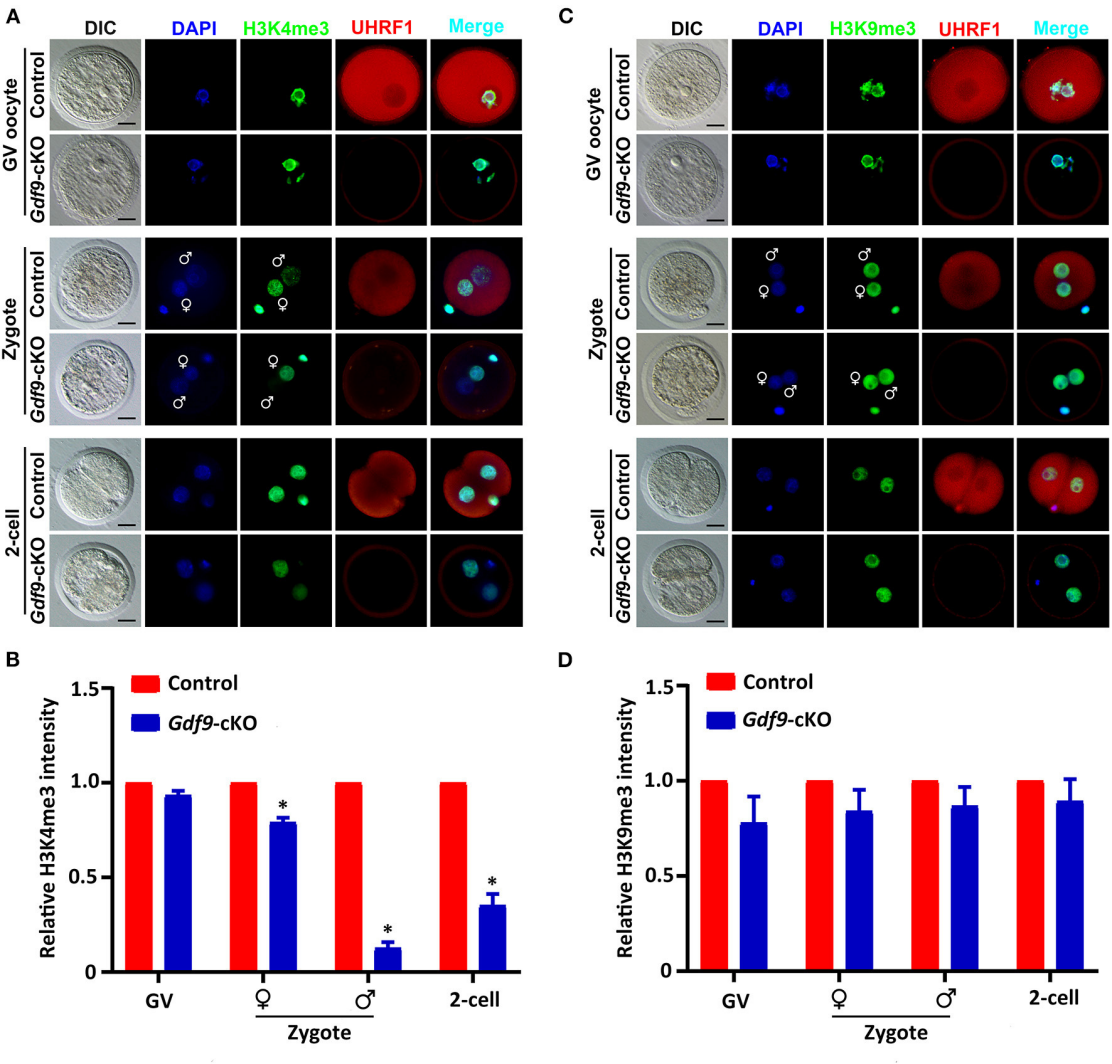
Maternal UHRF1 deficiency affects the H3K4me3 expression pattern but not H3K9me3 in zygote and two-cell embryos. **(A)** Immunofluorescent-staining using antibodies against H3K4me3 (in green) and UHRF1 (in red) at GV oocytes, zygotes, and two-cell embryos from control and *Gdf9*-cKO mice are shown. DNA was counterstained with DAPI (blue). Scale bar = 50 μm**. (B)** Quantification of the relative expression levels of H3K4me3 in control and *Gdf9*-cKO GV oocytes, zygotes, and two-cell embryos of **(A)**. Data are presented as mean ± SEM. *n* = 3 independent experiments (total number of GV oocytes analyzed: *n* = 80 for control, *n* = 75 for *Gdf9*-cKO; total number of zygotes analyzed: *n* = 123 for control, *n* = 106 for *Gdf9*-cKO; total number of two-cell embryos analyzed: *n* = 96 for control, *n* = 83 for *Gdf9*-cKO), *p < 0.05. **(C)** Immunofluorescent-staining using antibodies against H3K9me3 (in green) and UHRF1 (in red) at GV oocytes, zygotes, and two-cell embryos from control and *Gdf9*-cKO mice are shown. DNA was counterstained with DAPI (blue). Scale bar = 50 μm. **(D)** Quantification of the relative expression levels of H3K9me3 in control and *Gdf9*-cKO GV oocytes (total number analyzed: *n* = 89 for control, *n* = 92 for *Gdf9*-cKO), zygotes (total number analyzed: *n* = 135 for control, *n* = 131 for *Gdf9*-cKO), and two-cell embryos (total number analyzed: *n* = 125 for control, *n* = 93 for *Gdf9*-cKO) of **(C)**.

To determine whether maternal UHRF1 plays a critical role in histone modifications during oocyte and early embryo development, we next examined the H3K4 and H3K9 methylation levels in GV oocytes and early embryos from control and *Gdf9*-cKO mice using immunofluorescence assays. We found that the fluorescence signal of both H3K4me3 and H3K9me3 at GV oocytes did not change significantly in *Gdf9*-cKO mice compared with those in the control mice (Figures 3A,B). Interestingly, the overall H3K4me3 levels decreased significantly in *Gdf9*-cKO zygotes and two-cell stage embryos compared to that of controls, while the H3K9me3 levels did not significantly change in *Gdf9*-cKO embryos (Figures 3C,D). Additionally, the H3K4me3 mark was more severely reduced in the male pronucleus than in the female pronucleus in *Gdf9*-cKO zygotes (Figure 3B), suggesting that maternal UHRF1 plays an irreplaceable role in paternal H3K4me3 reprogramming in zygotes. Altogether, these observations show that the absence of maternal UHRF1 protein results in decreased levels of H3K4me3 in both parental genomes at the zygotes stage and genomes in two-cell embryos, and suggest that UHRF1 might be engaged with other chromatin modifiers to control global H3K4me3 marks after fertilization and early embryonic development.

### Absence of UHRF1 Abrogates the Normal Changes in Transcriptome by the Two-Cell Stage

**Figure 4:**
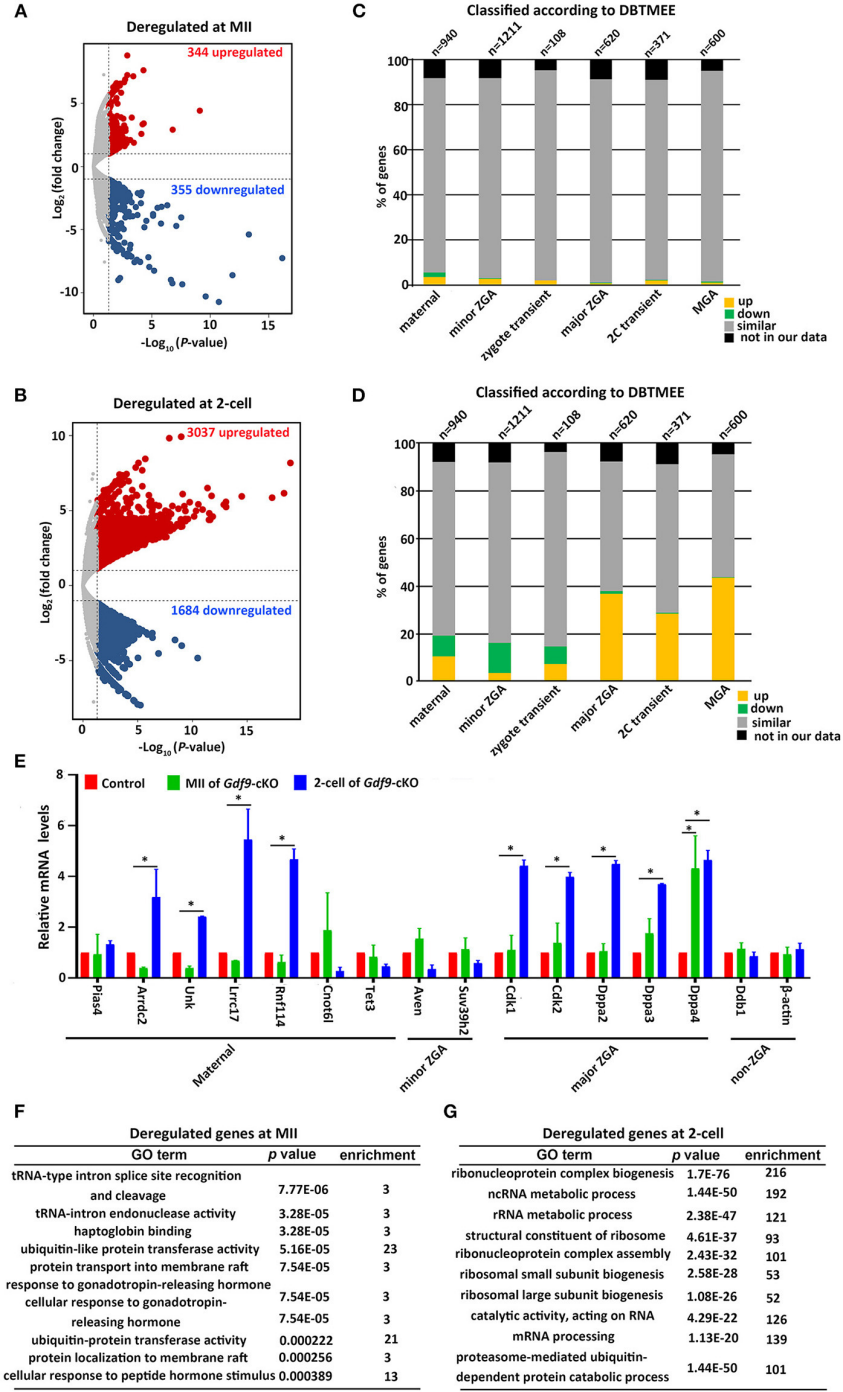
Abnormal ZGA upon absence of maternal UHRF1 revealed by transcriptome analysis. **(A,B)** Volcano plots showing the RNA-seq data obtained from **(A)** MII oocytes and **(B)** two-cell embryos of control and *Gdf9*-cKO mice. Red and dark blue dots indicate genes that were significantly upregulated and downregulated, respectively (fold change > 2, and FDR < 0.05) in *Gdf9*-cKO oocytes or embryos. **(C,D)** RNA-seq data from **(C)** MII oocytes or **(D)** two-cell embryos in comparison with the different categories of the gene catalog available at the Database of Transcriptome in Mouse Early Embryos (DBTMEE) generated the transcriptome analysis. The total number of genes in each class and found in this study is indicated on top of the graph. **(E)** RT-qPCR validates the expression of select upregulated and downregulated genes in MII oocytes and two-cell embryos from control and *Gdf9*-cKO mice. *p < 0.05 by Student's *t* test. **(F,G)** Top 10 representative GO terms (biological functions) enriched in *Gdf9*-cKO **(F)** MII oocytes and **(G)** two-cell embryos. *p* value indicates the significance of the enrichment.

As maternal UHRF1 deficiency in primordial oocytes arrests zygotic development, we next examined the potential effects of maternal UHRF1 on the transcriptome during zygotic gene activation. We thus subjected MII oocytes and two-cell embryos derived from WT and *Gdf9*-cKO mice for global RNA-seq analyses. Gene expression levels were assessed as fragments per kilobase of transcript per million mapped reads (FPKM), and DEseq was used as a normalization method across our samples to determine the relative gene expression between controls and mutants. At the MII stage, our analyses showed that 344 genes were upregulated and 355 genes were downregulated in the *Gdf9*-cKO oocytes when compared to those in WT oocytes (Figure 4A and Supplementary Table 3). Moreover, RNA-seq revealed a large number of upregulated (*n* = 3037, fold change > 2, *p* value < 0.05) or downregulated (*n* = 1684, fold change > 2, *p* value < 0.05) genes in the *Gdf9*-cKO two-cell embryos compared to those in WT embryos (Figure 4B and Supplementary Table 4). Together, RNA-seq data revealed that the normal transcriptomes were destroyed more severely in two-cell stage embryos than in MII oocytes upon maternal UHRF1 loss, suggesting that the absence of maternal UHRF1 most likely leads to transcriptome changes and severe defects after ZGA at the two-cell stage.

To further explore the effects of maternal UHRF1 deficiency on zygotic gene activation by the two-cell stage, we next assessed the deregulated genes in *Gdf9*-cKO MII oocytes and two-cell stage RNA-seq data according to the recent database of transcriptome in mouse early embryos (DBTMEE) (Park et al., 2013). DBTMEE was established by large-scale whole transcriptome analysis of preimplantation embryos, in which the genes are usually divided into six categories according to the transcriptional waves they expressed (Park et al., 2013; Ancelin et al., 2016). As shown in Figures 4C,D, we assessed the percentage of genes of each of our classes (up, down, not significantly changed, and not in our data) that overlapped with the different DBTMEE categories of transcription switches from the oocyte to the two-cell stage. Interestingly, we found that most of the deregulated genes in *Gdf9*-cKO MII oocytes did not change when mapped to the categories of DBTMEE, and only 31 and 30 upregulated genes belong to the genes annotated as maternal (3.3%) and minor ZGA genes (2.5%), respectively (Figure 4C and Supplementary Table 5). Strikingly, the downregulated genes in the *Gdf9*-cKO two-cell stage embryos fell essentially into the earliest stages and belonged to genes annotated as maternal (8.6%), minor ZGA genes (12.6%), and zygotic-transient (7.4%) (Figure 4D and Supplementary Table 6). In contrast, the majority of upregulated genes in the *Gdf9*-cKO two-cell embryos belonged to nearly four annotated genes, including maternal genes (10.6%), major ZGA (36.8%), two-cell transient (28.6%), and MGA (mid zygotic gene activation) (43.7%) (Figure 4D and Supplementary Table 6). These results suggest that the absence of maternal UHRF1 compromises gene expression in the ZGA at the two-cell stage.

To validate the RNA-seq data and analysis performed, we selected a set of genes with characteristic expression profiles, including maternal (*Pias4, Arrdc2, Unk, Lrrc17, Rnf114, Cnot6l*, and *Tet3*), minor ZGA (*Aven, Suv39h2*), major ZGA (*Cdk1, Cdk2, Dppa2, Dppa3*, and *Dppa4*), and non-ZGA (*Ddb1* and *Actb*) genes to perform RT-qPCR in control and *Gdf9*-cKO oocytes and two-cell embryos (Figure 4E). As predicted from RNA-seq results, the maternal and minor ZGA genes did not change significantly in MII oocytes of *Gdf9*-cKO mice. In contrast, nearly all maternal and major ZGA genes were significantly increased in two-cell embryos of *Gdf9*-cKO mice (Figure 4E). No difference in expression of *Pias4, Arrdc2, Unk, Lrrc17, Rnf114, Cnot6l, Tet3, Suv39h2, Cdk1/2*, and *Dppa2/3* was observed between controls and mutants at the MII oocyte stage, implying that the maternal pool of these mRNAs was not affected by maternal UHRF1 depletion. We next explored the link between chromatin modification and an increase in the transcription of major ZGA genes in maternal UHRF1-deficient embryos. Since the mouse ESC (embryonic stem cell) population resembles two-cell stage embryos and is pluripotent (Rodriguez-Terrones et al., 2018), we utilized the GSE113915 dataset and conducted a comparative analysis with ChIP-seq data for H3K4me3, H3K9me3, and UHRF1 enrichment between control and *Uhrf1* KO ESCs. We found that the histone modification landscape of representative genes related to the major ZGA genes (*Cdk1, Cdk2, Dppa2, Dppa3*, and *Dppa4*) significantly reduced H3K4me3 and H3K9me3 enrichment in *Uhrf1* KO ESCs compared with those in control (Supplementary Figures 2A–E), whereas the enrichment of H3K4me3 and H3K9me3 in the representative non-ZGA genes (*Ddb1* and *Actb*) was comparable between *Uhrf1* KO ESCs and WT ESCs (Supplementary Figures 2F,G). Furthermore, H3K9me3 enrichment was significantly lower than that of H3K4me3 in the representative major ZGA genes in mouse ESCs (Supplementary Figure 2). We also found that UHRF1 was marginally enriched in *Dppa2, Dppa3, and Dppa4* genes in WT ESCs (Supplementary Figures 2C–E). Moreover, we examined the *Cdk1, Cdk2, Dppa2, Dppa3*, and *Dppa4* expression in MII oocytes, zygotes, and two-cell embryos derived from control and *Gdf9*-cKO mice and found that the mRNA levels of *Cdk1, Cdk2, Dppa2*, and *Dppa4*, but not *Dppa3*, increased at the zygote stage (Supplementary Figure 2H), further supporting that ZGA genes were activated in *Gdf9*-cKO embryos. Thus, together with the RNA-seq data, these bioinformatic results suggest that maternal UHRF1 deficiency compromises the gene expression in ZGA at the two-cell stage to some extent and there is a link between chromatin modification and an increase in the transcription of major ZGA genes in maternal UHRF1 mutant embryos.

A further GO analysis of the DEGs in MII oocytes revealed that the DEGs in tRNA-type intron splice and cleavage, ubiquitin-like protein transferase activity, and protein transport were significantly enriched (Figure 4F). GO analysis of the deregulated genes from two-cell stage embryos is implicated in ribosome biogenesis, ncRNA/rRNA metabolic process, and mRNA processing (Figure 4G). Collectively, these results suggest that maternal UHRF1 is necessary for the transcriptional regulation of specific genetic pathways implicated in fundamental biological functions such as RNA metabolic process, protein transport, and ribosome biogenesis regulation. These combined defects could be consistent with the inability of the mutant embryos to develop beyond the two-cell stage.

### Maternal UHRF1 Deficiency Induces the Transposable Elements (TEs) Derepression and DNA Damage

**Figure 5:**
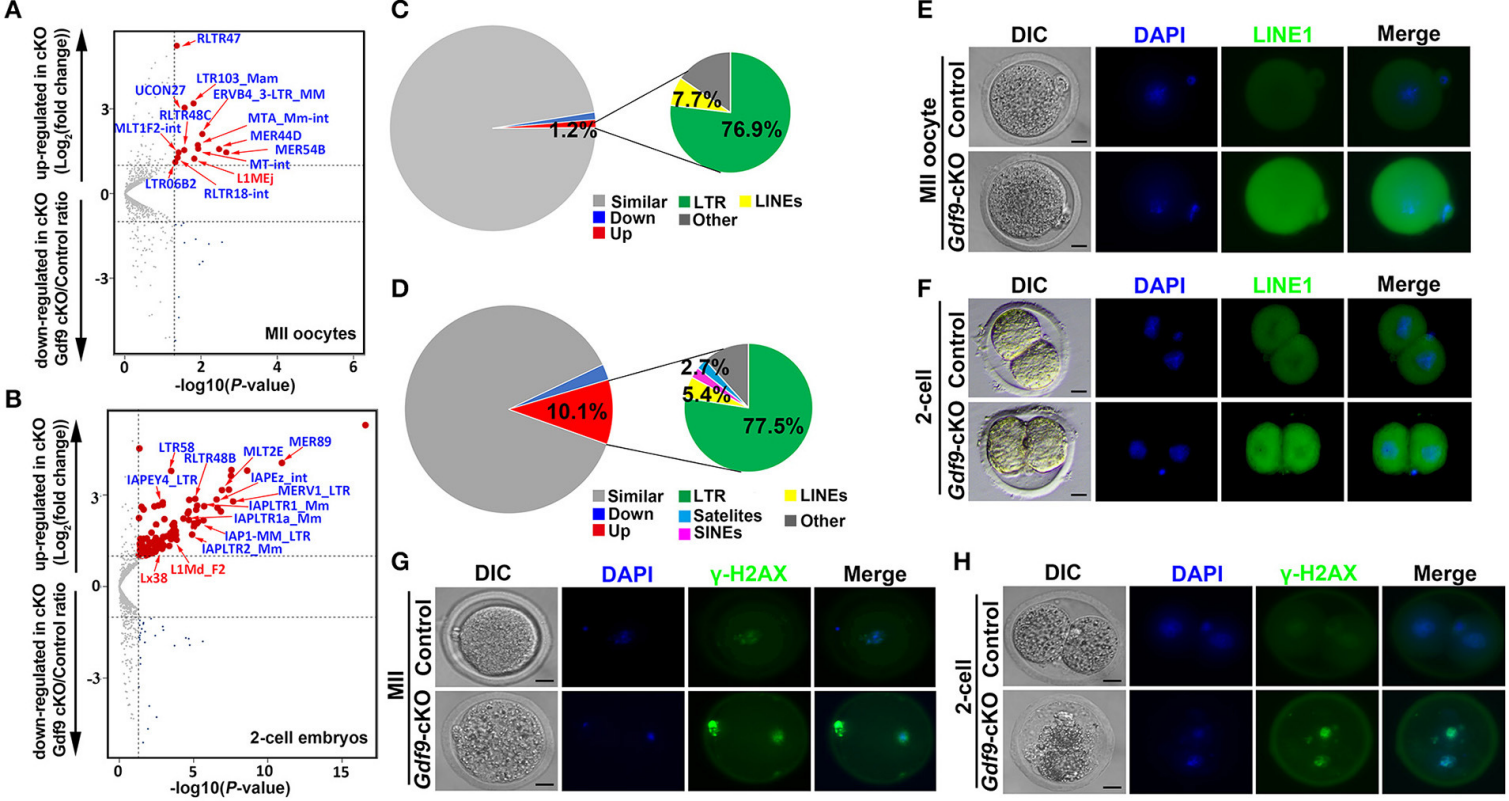
Increased retrotransposons and γ-H2AX in MII oocytes and two-cell embryos depleted for maternal UHRF1. **(A,B)** Volcano plots depicting activation of individual retrotransposon repetitive elements at **(A)** MII oocytes and **(B)** two-cell embryos of *Gdf9*-cKO mice. Red and dark blue dots indicate repeat classes with significantly increased and decreased expression, respectively, in *Gdf9*-cKO compared with those in controls. **(C,D)** Pie chart representing the percentage of each category of repeat elements analyzed in **(C)** MII oocytes and **(D)** two-cell embryos RNA-seq data. **(E,F)** The representative immunofluorescent staining images for anti-LINE1 Orf1 at **(E)** MII oocytes and **(F)** two-cell embryos from control and *Gdf9*-cKO mice are shown. Nuclei were stained with DAPI. Scale bar = 50 μm. **(G,H)** Immunostaining images using antibodies directed against γ-H2AX (a marker of DNA strand break) for **(G)** MII oocytes and **(H)** two-cell embryos from control and *Gdf9*-cKO are shown. Nuclei were stained with DAPI. Scale bar = 50 μm.

Many TEs are known to be expressed in early mouse embryos, and some of them might even be essential for new events of retrotransposition between fertilization and implantation (Peaston et al., 2004; Kano et al., 2009; Fadloun et al., 2013). During early embryonic development, the repression of some of these TEs is correlated with the expression of active chromatin marks such as H3K4me3 (Fadloun et al., 2013). Since maternal UHRF1 depletion significantly decreases H3K4me3 expression levels in zygotes and two-cell embryos (Figures 3A,B) and UHRF1 deletion in male germ cells results in activation of retrotransposons (Dong et al., 2019), we explored whether the absence of maternal UHRF1 affects repetitive element expression in oocytes and early embryos. Thus, we investigated the RNA-seq data from control and *Gdf9-*cKO oocytes and two-cell embryos for the relative expression of repetitive elements. As shown in Figures 5A,B, compared with the control groups, 13 and 111 repetitive elements were significantly upregulated in *Gdf9*-cKO MII oocytes and two-cell embryos, respectively (fold change > 2, *p* < 0.05, Wald test: Supplementary Tables 7, 8), indicating maternal UHRF1 deletion results in abnormal TEs activation in both oocytes and two-cell embryos. Interestingly, we found that most of the upregulated TEs were long terminal repeat (LTR) elements in *Gdf9*-cKO, which account for 76.9% and 77.5% at MII oocytes and two-cell embryos, respectively (Figures 5C,D). However, we found that the expression levels of L1MEj at MII oocytes and L1Md_F2, L1Md_A, L1_Mus1, and L1MEi at two-cell embryos increased three- to sixfold (Supplementary Tables 7, 8), suggesting that LINE-1 family was activated in *Gdf9*-cKO oocytes and embryos. To assess whether the observed increase in LINE-1 transcription might correspond to full-length LINE-1 protein, we performed the immunofluorescence (IF) to detect the expression of ORF1, one of the two LINE-1 encoded proteins. As expected, we found that the fluorescence intensity increased in both *Gdf9-*cKO MII oocytes and two-cell embryos compared with those in controls (Figures 5E,F). These results suggest that LINE-1 deregulation observed at the RNA level might indeed lead to the increased levels of LINE-1 ORF1 proteins. We next investigated whether the overexpression of such TEs is harmful for genome integrity. Therefore, we performed IF staining for γ-H2A.X, a marker of DNA damage response, to assess whether increased DNA damage signaling is seen in *Gdf9*-cKO oocytes and embryos. Indeed, we found that γ-H2A.X was more strongly stained in mutant MII oocytes and two-cell embryos than that in controls (Figures 5G,H). Taken together, these results suggest that maternal UHRF1 is essential for repetitive element repression and genome integrity of oocytes and early embryos.

## Discussion

Our previous study showed that UHRF1, an epigenetic factor, is essential for suppressing the retrotransposons and interacting with the piRNA pathway in male germ cells (Dong et al., 2019). Although the effects of maternal *Uhrf1* mutant in oocytes have been assessed (Maenohara et al., 2017; Cao et al., 2019), the biological function of such changes and the identification of the histone modification involved in early embryonic development during MZT remains to be identified. In the present study, we focused on the function of UHRF1 in the dynamic development process of oocytes to embryos after fertilization. We showed that maternal UHRF1 deficiency in oocytes resulted in abnormal oocytes in the MII stage and arrested the development of fertilized eggs at the two-cell stage, some of which have also been reported in previously published studies (Maenohara et al., 2017; Cao et al., 2019). Furthermore, this study revealed that UHRF1 is essential for early post-zygotic embryo development as a critical regulator of the dynamic balance of maternal and zygote transcript pools, and maternal UHRF1 deficiency in primordial oocytes resulted in embryo developmental arrest at the two-cell stage. We further showed that maternal UHRF1 is a regulator that controls histone H3K4me3 marks at the zygote and two-cell stages, and maternal UHRF1 is also necessary for the appropriate repression of some transposon elements, particularly the LTR and LINE-1 subfamilies.

It is worth mentioning that, in the present study, we found that maternal UHRF1 deficiency decreases the global DNA methylation levels in GV oocytes and zygotes, which has also been reported in previously published studies (Maenohara et al., 2017; Cao et al., 2019). These observations are largely supported by the function of UHRF1 in DNA methylation maintenance in mammalian cells (Bostick et al., 2007). UHRF1 is also essential for regulating heterochromatin formation and associated with histone modifications such as H3K9me3 and H3K4me3 marks in somatic and germ cells (Xie et al., 2012; Dong et al., 2019). Notably, the H3K4me1/2/3 expression levels are associated with the development of the zygote to the two-cell embryo stage (Shao et al., 2014). H3K4me3 is linked to active vs. inactive regions of the genome and is crucial for the accumulation of maternal factors and MZT during oogenesis and preimplantation embryogenesis (Bultman et al., 2006; Macfarlan et al., 2011; Aoshima et al., 2015; Ancelin et al., 2016; Yu et al., 2017; Hanna et al., 2018; Sankar et al., 2020). The deletion of maternal CFP1 decreases H3K4me3 expression in oocytes and further leads to decreased developmental competence and defects in the MZT (Yu et al., 2017).

Maternal UHRF1 participates in global epigenetic reprogramming of oocytes and preimplantation embryos (Maenohara et al., 2017). Upon fertilization, global H3K4me3 on the paternal allele of the zygote undergoes extensive reprogramming, and paternal H3K4me3 peaks are weak in zygotes and then reappear particularly at the late two-cell stage (Zhang et al., 2016). Interestingly, in the present study, we found that maternal UHRF1 depletion in primordial oocytes reduced the expression of H3K4me3 in two-cell embryos instead of MII oocytes, but did not affect the H3K9me3 expression levels. We thus speculated that maternal UHRF1 plays an irreplaceable role in paternal H3K4me3 reprogramming in zygotes and more significantly reduced H3K4me3 in male pronucleus than that in female pronucleus in *Gdf9*-cKO mice. In comparison, the global H3K9me2 levels decreased in GV and MII oocytes from *Zp3-*cKO mice, without impacting the H3K4me3, H3K9me3, H3K36me3, H3K9ac, H3K27ac, and H4K8ac levels (Cao et al., 2019). In addition, UHRF1 recruits H3K9 methyltransferase that catalyzes the bi-and tri-methylation of H3K9 (Kim et al., 2018). Therefore, the variance change of H3K9me3 in oocytes, embryos, and ESCs of UHRF1 mutant mice indicated that the effects of UHRF1 deficiency may be histone-locus-specific and cellular specific.

Chromatin-based repression is imperative for regulating ZGA and transition from the zygote to the two-cell or four-cell stage (Nothias et al., 1995; Wiekowski et al., 1997; Ma et al., 2001; Ma and Schultz, 2008). Both KDM1A and KDM4A deficiency lead to the decrease or loss of the genes required for the transition toward the two-cell embryo stage (Ancelin et al., 2016; Sankar et al., 2020). Although the decrease of H3K4 methylation levels occurred in maternal UHRF1 mutant zygotes and two-cell embryos, we found an increase of the expression levels of the genes required for the ZGA (such as in the major ZGA, two-cell transient, and MGA waves). Thus, UHRF1-deficient embryos might possess deficits other than the histone modification. Additionally, the transcription factors developmental pluripotency-associated 2 (*Dppa2*) and *Dppa4* are necessary to activate the two-cell genes, particularly the transcription factor double homeobox (DUX) (De Iaco et al., 2019; Eckersley-Maslin et al., 2019). Similarly, compared with the controls, we found that the two-cell embryos of maternal UHRF1 mutant showed higher transcriptional activity of DPPA2 and DPPA4, which might be the main reason for gene activation at the major ZGA, 2C transient, and MGA waves. Furthermore, oocytes that lack *Dppa3* (*Stella*) acquire excessive DNA methylation at the genome-wide level and induce UHRF1 ectopic nuclear accumulation (Payer et al., 2003; Li et al., 2018). Meanwhile, UHRF1 deficiency in the two-cell embryos resulted in increased transcriptional levels of *Dppa3* and global DNA hypomethylation (Li et al., 2018). Combined with the results of the database analysis, UHRF1 was marginally enriched in *Dppa2, Dppa3*, and *Dppa4* genes in WT ESCs, of which enrichment revealed a marked reduction of H3K4me3 in *Uhrf1* KO ESCs compared with controls. Thus, we could not exclude the possibility that UHRF1 and DPPAs (DPPA2, DPPA3, and DPPA4) had mutual restriction and interdependence function during the oocyte and preimplantation embryo development.

Another exciting finding of the current study is that we found that the repetitive elements, especially LTR and LINE-1, were not silenced correctly in maternal UHRF1-deficient oocytes and two-cell embryos. This observation is consistent with the function of UHRF1 in other types of cells, such as male germ cells (Dong et al., 2019) and neural stem cells (Ramesh et al., 2016), which added a layer of UHRF1 function in female germ cells and further supported the notion that UHRF1 maintains the genome integrity in both somatic and germ cells by silencing repetitive elements. Retrotransposon activity is suppressed by multiple mechanisms, including small non-coding piRNAs, DNA methylation, and repressive histone modifications in mammalian germ cells (Yang and Wang, 2016). The analysis of CG methylation revealed that the absence of maternal UHRF1 caused a proportional decrease in CG methylation of repetitive elements, including LINE1 and IAPs, in full-grown oocytes and blastocysts (Maenohara et al., 2017). Furthermore, these results, together with H3K4me3 decrease, are consistent with the context that H3K4me3 loss at LINE1 elements without a change in H3K9 methylation (Fadloun et al., 2013) might activate retrotransposon expression in oocytes and preimplantation embryos. As expected, we detected LINE-1 and LTR activation in the *Uhrf1* mutant MII oocytes and a significant increase in LINE-1 and LTR expression and LINE-1 ORF1 protein levels in the *Uhrf1* mutant two-cell embryos. In addition, UHRF1 loss in neural stem cells induced retrotransposon activation followed by global genomic DNA hypomethylation (Ramesh et al., 2016). Thus, the failure of retrotransposon suppression in UHRF1 mutant embryos in the present study might also be caused by DNA hypomethylation and abnormal histone modifications. Collectively, we speculated that maternal UHRF1 protein has a potential role in histone-based and DNA methylation defense mechanisms to safeguard the genome from retrotransposition during oocytes and preimplantation development. However, the mechanism and molecular links through which UHRF1 is recruited to the repetitive elements to suppress retrotransposons during the maternal–zygotic transition remain to be elucidated.

In summary, our study demonstrated that maternal UHRF1 participates in controlling genomic DNA methylation, H3K4me3 modification, and the transcription of mouse maternal and zygote mRNAs in early embryos, providing them with high developmental competence after fertilization. A further intriguing concept in this study revealed that UHRF1 is essential for elevating developmental competence in MZT. We believe that our findings provide a novel insight into epigenetic regulation in early embryo development and a basis for improving assisted reproductive technologies.

## Data Availability Statement

The datasets generated for this study can be found in online repositories. The names of the repository/repositories and accession number(s) can be found in the article/Supplementary Material.

## Ethics Statement

The animal study was reviewed and approved by Institutional Animal Care and Use Committee of Tongji Medical College, Huazhong University of Science and Technology.

## Author Contributions

YW, JD, and SF conceived and designed the research. YW, JD, SF, QZ, PD, MX, YW, CL, and XW performed all experiments and data analyses. YW, JD, and SF wrote the manuscript. SY revised the manuscript and supervised the project. All authors read and approved the manuscript.

## Conflict of Interest

The authors declare that the research was conducted in the absence of any commercial or financial relationships that could be construed as a potential conflict of interest.

## Supplementary Material

Supplementary Figure 1 Generation of conditional knockout of UHRF1 mouse models in primordial or growing oocytes. **(A)** The schematic illustration of *Gdf9*-Cre or *Zp3*-Cre-mediated *Uhrf1* knockout in oocytes from primordial follicle or growing follicles is shown. **(B)** The strategy of generation of specific UHRF1 knockout mouse models is shown. **(C)** RT-qPCR analyses show that *Uhrf1* mRNA was nearly undetectable in *Gdf9*-cKO GV oocytes. ^***^*p* < 0.001 by Student's *t* test. **(D)** Western blotting shows UHRF1 protein was undetectable in *Gdf9*-cKO GV oocytes. GAPDH served as a loading control. **(E)** Immunohistochemical staining (IHC) results show the UHRF1 protein expression in different follicles of control and *Gdf9*-cKO mice. Scale bar = 50 μm. For each genotype, at least five females were used. **(F)** The representative immunofluorescent staining images for anti-UHRF1 in GV oocytes and different stage of preimplantation embryos from control and *Gdf9*-cKO mice. Scale bar = 10 μm. **(G–H)** RT-qPCR analyses and western blotting show *Uhrf1* **(G)** mRNA and **(H)** protein were nearly undetectable in *Zp3*-cKO GV oocytes. ^***^*p* < 0.001. GAPDH served as a loading control.

Supplementary Figure 2 ChIP-Seq revealed the histone modification changes of Dppas genes between *Uhrf1* KO and WT ESCs. (**A–E**) Genome browser panels showing read coverage at **(A)** *Cdk1*, **(B)** *Cdk2*, (C) *Dppa2*, **(D)** Dppa3, and **(E)** *Dppa4* from UHRF1, H3K9me3, and H3K4me3 ChIP-seq of WT and *Uhrf1* KO ESCs. **(F)** The histogram shows the representative gene expression (*Cdk1, Cdk2, Dppa2, Dppa3*, and *Dppa4*) of the major ZGA at MII oocytes, zygotes, and two-cell embryos from control and *Gdf9*-cKO mice. ^*^*p* < 0.05 by Student's *t* test; ns, no significant.
