# Supplementary figures and images for: Maternal UHRF1 Is Essential for Transcription Landscapes and Repression of Repetitive Elements During the Maternal-to-Zygotic Transition
Source: Front Cell Dev Biol. 2021 Feb 9;8:610773. doi: 10.3389/fcell.2020.610773 (PMC7902027; doi:10.3389/fcell.2020.610773)

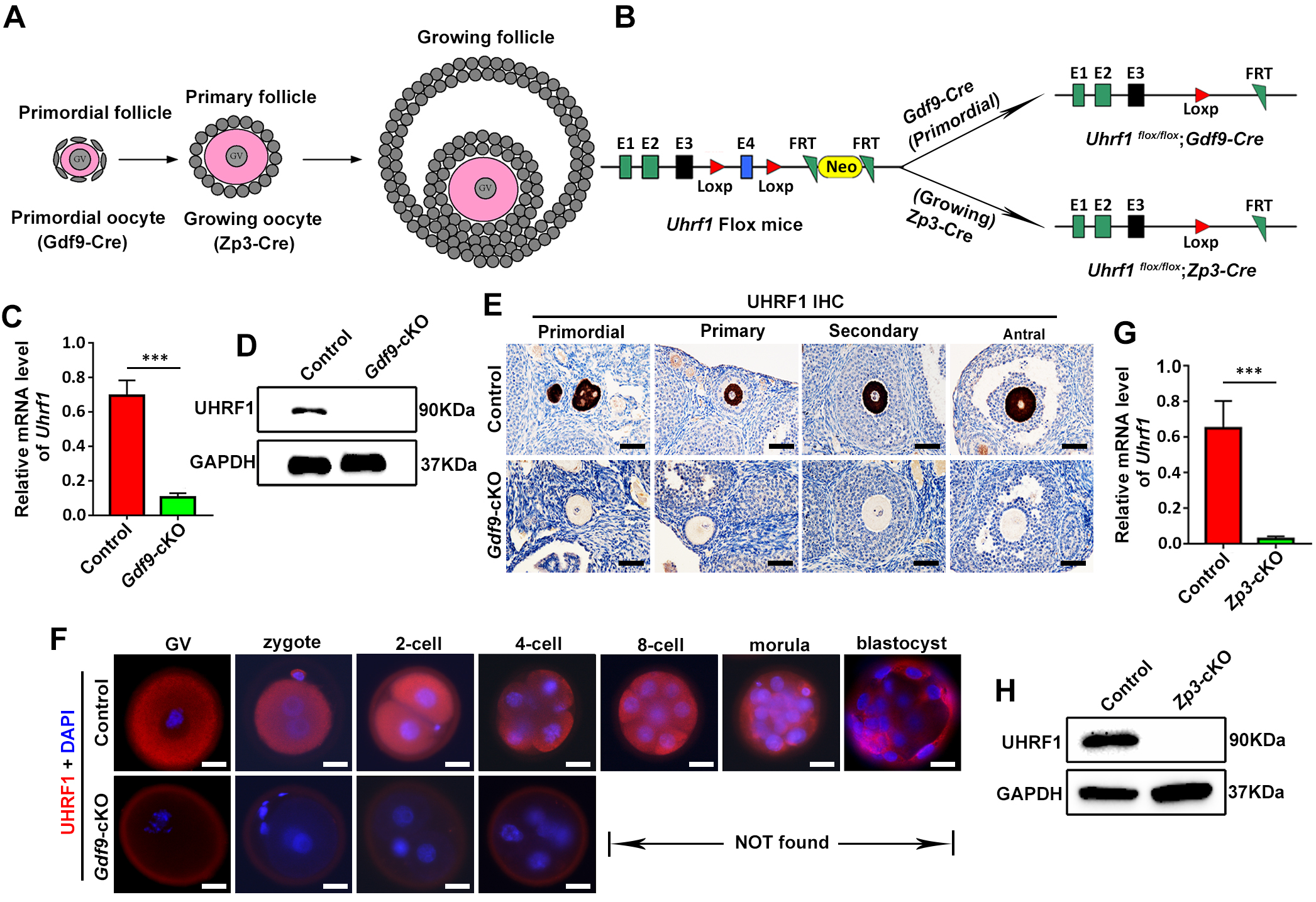

Supplement: Supplementary Figure 1 — Generation of conditional knockout of UHRF1 mouse models in primordial or growing oocytes. (A) The schematic illustration of Gdf9-Cre or Zp3-Cre-mediated Uhrf1 knockout in oocytes from primordial follicle or growing follicles is shown. (B) The strategy of generation of specific UHRF1 knockout mouse models is shown. (C) RT-qPCR analyses show that Uhrf1 mRNA was nearly undetectable in Gdf9-cKO GV oocytes. ***p < 0.001 by Student's t test. (D) Western blotting shows UHRF1 protein was undetectable in Gdf9-cKO GV oocytes. GAPDH served as a loading control. (E) Immunohistochemical staining (IHC) results show the UHRF1 protein expression in different follicles of control and Gdf9-cKO mice. Scale bar = 50 μm. For each genotype, at least five females were used. (F) The representative immunofluorescent staining images for anti-UHRF1 in GV oocytes and different stage of preimplantation embryos from control and Gdf9-cKO mice. Scale bar = 10 μm. (G–H) RT-qPCR analyses and western blotting show Uhrf1 (G) mRNA and (H) protein were nearly undetectable in Zp3-cKO GV oocytes. ***p < 0.001. GAPDH served as a loading control. [file Image_1.JPEG]

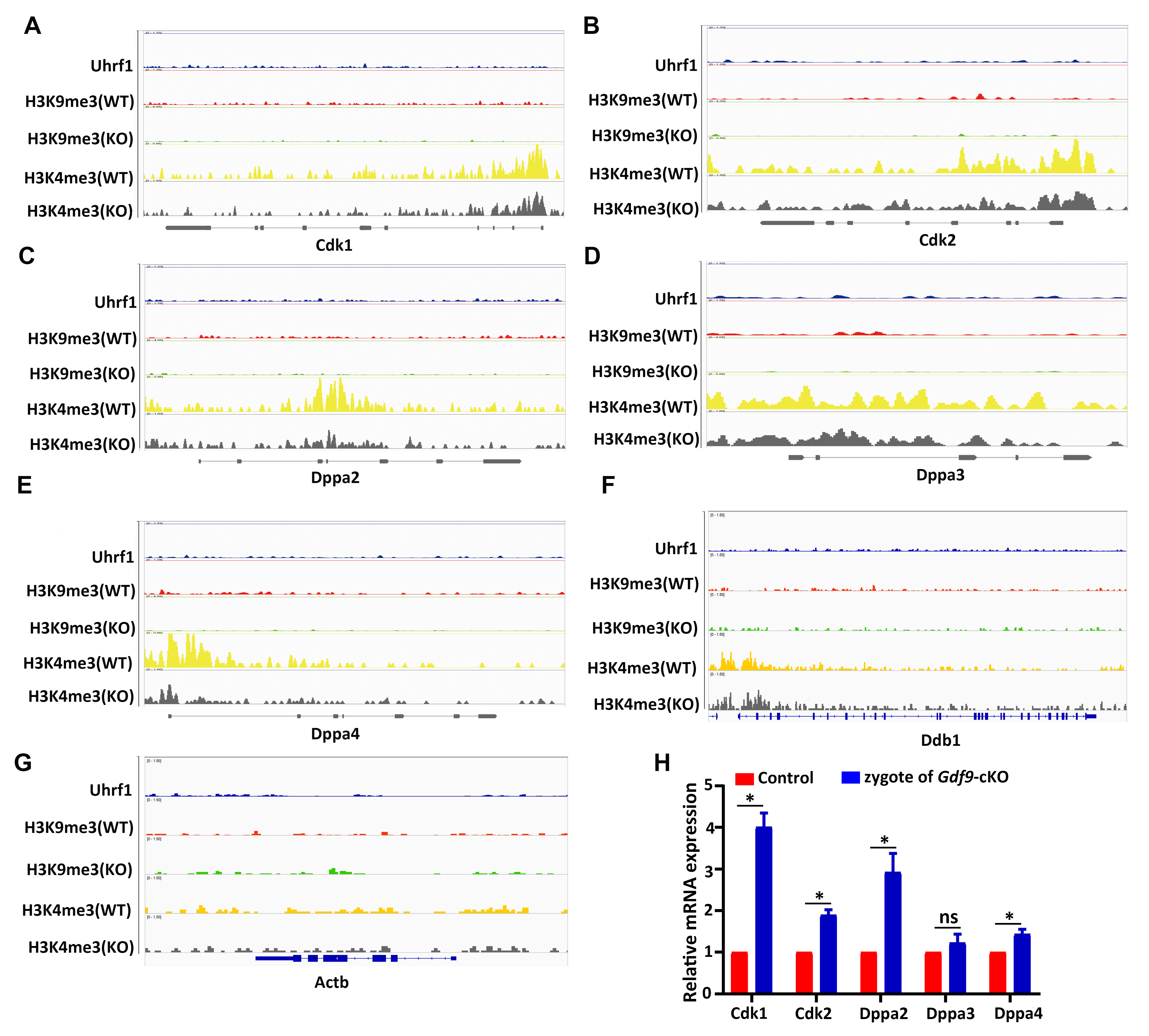

Supplement: Supplementary Figure 2 — ChIP-Seq revealed the histone modification changes of Dppas genes between Uhrf1 KO and WT ESCs. (A–E) Genome browser panels showing read coverage at (A) Cdk1, (B) Cdk2, (C) Dppa2, (D) Dppa3, and (E) Dppa4 from UHRF1, H3K9me3, and H3K4me3 ChIP-seq of WT and Uhrf1 KO ESCs. (F) The histogram shows the representative gene expression (Cdk1, Cdk2, Dppa2, Dppa3, and Dppa4) of the major ZGA at MII oocytes, zygotes, and two-cell embryos from control and Gdf9-cKO mice. *p < 0.05 by Student's t test; ns, no significant. [file Image_2.JPEG]
