# Supplementary material for: Maternal UHRF1 Is Essential for Transcription Landscapes and Repression of Repetitive Elements During the Maternal-to-Zygotic Transition
Source: Front Cell Dev Biol. 2021 Feb 9;8:610773. doi: 10.3389/fcell.2020.610773 (PMC7902027; doi:10.3389/fcell.2020.610773)
Supplement: Supplementary file 7 [file Table_5.DOCX]

**Table S3.** Comparing the MII stage transcriptome of the Uhrf1 mutant oocytes to DBTMEE. The numbers showed the genes for comparing our two-cell stage RNA-seq data with the different categories for the gene catalog found in DBTMEE. Total genes considered = 3552 and total genes changed = 109 (3.1%). Our dataset covers the genes categorized on the public resource with a minimum of 92% of genes.

|  | Up | Down | Similar | Not in our data | Total in DBTMEE |
| --- | --- | --- | --- | --- | --- |
| maternal | 31 | 20 | 812 | 77 | 940 |
| Minor ZGA | 30 | 5 | 1076 | 100 | 1211 |
| 1C transient | 2 | 0 | 101 | 5 | 108 |
| major ZGA | 3 | 3 | 561 | 54 | 620 |
| 2C transient | 7 | 1 | 330 | 33 | 371 |
| MGA | 5 | 3 | 563 | 29 | 600 |
