# Supplementary material for: Maternal UHRF1 Is Essential for Transcription Landscapes and Repression of Repetitive Elements During the Maternal-to-Zygotic Transition
Source: Front Cell Dev Biol. 2021 Feb 9;8:610773. doi: 10.3389/fcell.2020.610773 (PMC7902027; doi:10.3389/fcell.2020.610773)
Supplement: Supplementary file 8 [file Table_6.DOCX]

**Table S4**. Comparing the two-cell stage transcriptome of the Uhrf1 mutant embryos to DBTMEE. The numbers showed the genes for comparing our two-cell stage RNA-seq data with the different categories for the gene catalog found in DBTMEE. Total genes considered = 3552 and total genes changed = 1000 (28%). Our dataset covers the genes categorized on the public resource with a minimum of 92% of genes.

|  | Up | Down | Similar | Not in our data | Total in DBTMEE |
| --- | --- | --- | --- | --- | --- |
| maternal | 100 | 81 | 684 | 75 | 940 |
| Minor ZGA | 45 | 153 | 915 | 98 | 1211 |
| 1C transient | 8 | 8 | 88 | 4 | 108 |
| major ZGA | 228 | 7 | 337 | 48 | 620 |
| 2C transient | 106 | 1 | 231 | 33 | 371 |
| MGA | 262 | 1 | 309 | 28 | 600 |
